# Zero-Point Corrections for Isotropic Coupling Constants for Cyclohexadienyl Radical, C_6_H_7_ and C_6_H_6_Mu: Beyond the Bond Length Change Approximation

**DOI:** 10.3390/molecules18054906

**Published:** 2013-04-25

**Authors:** Bruce S. Hudson, Suzanne K. Chafetz

**Affiliations:** Department of Chemistry, Syracuse University, Syracuse, NY 13244-4100, USA

**Keywords:** muon, cyclohexadienyl, spin resonance, zero-point, hyperfine coupling, DFT

## Abstract

Zero-point vibrational level averaging for electron spin resonance (ESR) and muon spin resonance (µSR) hyperfine coupling constants (HFCCs) are computed for H and Mu isotopomers of the cyclohexadienyl radical. A local mode approximation previously developed for computation of the effect of replacement of H by D on ^13^C-NMR chemical shifts is used. DFT methods are used to compute the change in energy and HFCCs when the geometry is changed from the equilibrium values for the stretch and both bend degrees of freedom. This variation is then averaged over the probability distribution for each degree of freedom. The method is tested using data for the methylene group of C_6_H_7_, cyclohexadienyl radical and its Mu analog. Good agreement is found for the difference between the HFCCs for Mu and H of CHMu and that for H of CHMu and CH_2_ of the parent radical methylene group. All three of these HFCCs are the same in the absence of the zero point average, a one-parameter fit of the static HFCC, a(0), can be computed. That value, 45.2 Gauss, is compared to the results of several fixed geometry electronic structure computations. The HFCC values for the *ortho*, *meta* and *para* H atoms are then discussed.

## 1. Introduction

The lifetime of muonium (Mu), the hydrogenic species formed by an electron and a positive muon, is 2.2 µs [1], long enough for complete thermalization after addition to a double bond. Muon spin resonance (µSR) methods are useful for determination of hyperfine coupling constants (HFCCs) of radicals especially of highly reactive species [1,2,3,4]. This capability of µSR provides data for simple systems that are amenable to advanced electronic structure treatments. One of these systems is the cyclohexadienyl radical, C_6_H_7_, formed by addition of H or Mu to benzene (or removal of H from 1,4-cyclohexadiene) [5,6,7,8,9,10,11,12,13]. In making comparison between electronic structure theory and experiment there is a need to include zero point level effects. The isotope effect is the difference of in this effect for isotopic species. This work follows previous studies [14,15] in that the effect of this replacement on a single bond is treated. 

The effect of replacement of an H by Mu is usually attributed to a change in the C-X bond length [2,6,14,15]. This is also the long-standing explanation for the effect of replacement of H by D on the ^13^C-NMR spectrum of molecules [16]. In the ^13^C-NMR case this appears to be a reasonable approximation for the carbon that bears a deuterium. The shifts for the other carbons in the same monodeutero species are, however, not reproduced by this method until the bending degrees of freedom are added. These are the isotope effects that have information about the connectivity and geometry of the molecule. The attribution of the isotope effect to a bond length change for either ^13^C-NMR or μSR are, in each case, “isolated bond” or “local mode” approximations in the extreme limit in which the probability distribution is treated as a delta function at the average position. In a recent publication on the deuterium isotope effect on ^13^C-NMR spectra of hydrocarbons [16], we showed that inclusion of all three degrees of freedom, bend as well as stretch, and including the contraction of the stretching motion when D replaces H, results in good agreement for the substituted carbon and provides agreement for the smaller effects on neighboring carbons which diminishes as the number of intervening bonds increases. 

The stretch degree of freedom has a net isotope effect because isotopic substitution results in both the change in average position (“displacement”) and the change in width of the distribution for one isotopic form *vs.* the other. The variation of ^13^C chemical shift with increasing CH bond length, R, is found to be exactly fit by a second order polynomial. The linear part of the variation multiplies the displacement term while the quadratic variation picks out the change in the second moment of the probability distribution. For the NMR case there are no higher terms in the variation in the range of finite probability. This is not the case for the corresponding μSR calculations.

The deuterium isotope effect on ^13^C chemical shifts is small; at most 400 ppb compared to chemical shift differences of the order of 100 ppm. The smaller shifts of distant carbons in the range of −20 to +140 ppb are known to a precision of about 1 ppb. The quantity reported in these experiments is the difference in the chemical shifts with isotopic substitution. This means that the quantity being computed for comparison with the experiment is the difference of two calculations of the ^13^C chemical shift and thus is not appreciably dependent on the absolute precision of the method used for geometry optimization or NMR calculation. This also justifies the use of the isolated bond approximation in that the effect of the zero point motions of the H atoms in all the unsubstituted sites should have the same effect on the chemical shifts in both the H and D forms and so they cancel out in the difference. This appears to be justified by comparison of the resulting isotope effect based on the local mode approximation with ca. 100 high precision experimental results for rigid hydrocarbons. 

We have two objectives in this present work. One is the demonstration of the suitability of a transferable local mode DFT-based method for the computation of muon and hydrogen zero point level effects in µSR and ESR spectroscopies and their difference, the isotope effect. This task is performed in analogy to that used in the deuterium NMR calculations including all three degrees of freedom and, especially, the change in the stretch distribution width for the muon. The second objective is to establish a benchmark value for the geometry optimization and HFCC computation method. That is done by “correcting” the experimental values for the zero point effect to obtain a “target” a(0) value. 

The computation of the effect of zero point level motion on the ESR (or μSR) spectrum proceeds in the following steps. An optimized C_2v_ is displaced along the stretch and each bend motions for one of the two equivalent CH bonds. The resulting variation of the energy relative to the value at the minimum along the stretch displacement is converted to cm^−1^ and fit to a Morse potential. The in-plane and out-of-plane bending degrees of freedom are treated in the same fashion except the potential is fit to a polynomial which is found to be nearly harmonic.

**Figure 1:**
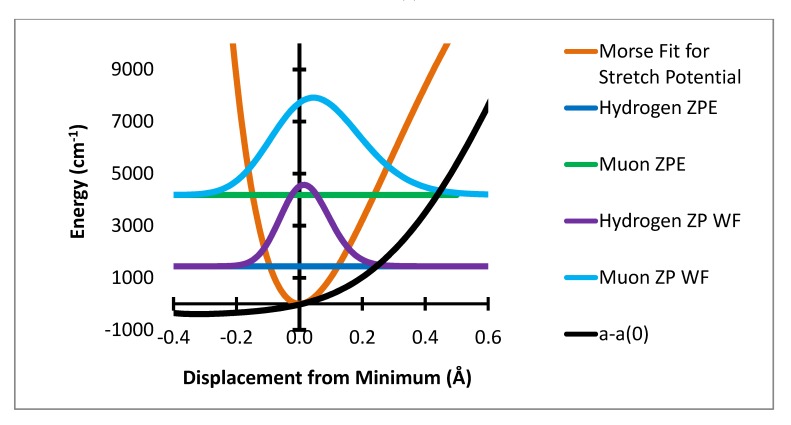
Stretching potential energy with zero point energy levels and probability distributions for H and Mu and variation of a-a(0) Gauss × 10^5^.

**Figure 2:**
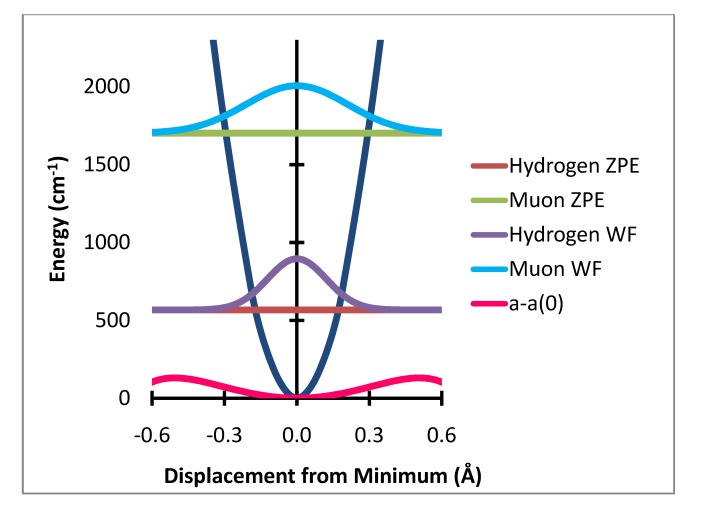
Out of plane bending potential with zero point energy levels and probability distributions for H and Mu and variation of a-a(0) Gauss × 10^4^.

**Figure 3:**
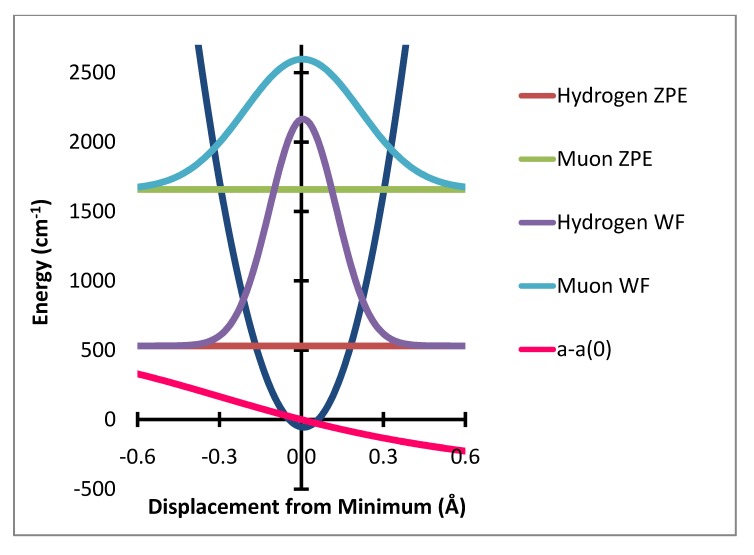
In-plane bending potential (dark blue) with zero point energy levels and probability distributions for H and Mu and variation of a–a(0) Gauss × 50,000.

The variation of the individual hyperfine coupling constants from the equilibrium value a(0) is then averaged over the populated zero point level geometries. This produces the zero point level averaged value of the difference between the hyperfine coupling at the equilibrium geometry, a(0), and that for the zero point level. These are shown in Table 1. The computational method used here is summarized in Figure 1, Figure 2 and Figure 3. Figure 1 shows the variation of the potential energy with stretching degree of freedom. Figure 2 and Figure 3 show the corresponding variation for the two bending degrees of freedom.

## 2. Results and Discussion

In this initial study we concentrate on the largest zero point effects which are measurable as isotope effects. For this discussion the methylene carbon C1 has attached H1 or Mu and H1'. H1 and H1' are identical in C_6_H_7_ but Mu and H1' differ in C_6_H_6_Mu. The isotope effects then are the difference in the measured HFCC for Mu (as A', *i.e.*, corrected for the magnetic moment ratio) and that for H1' and the difference between H1' of C_6_H_6_Mu and H1 of C_6_H_7_. In the comparison of the HFCC for Mu with that for H1' both values are from experiment for the gas phase. The C_6_H_7_ comparison is necessarily with data obtained in solution. These are then compared to the difference in the ZP corrections.

Table 1 contains the main results of this work. All entries are in Gauss. The computed contributions to the Zero Point Correction (ZPC) due to the stretching and two bending degrees of freedom are obtained by averaging the variation of the HFCC for the responding atom relative to that at the equilibrium geometry, a-a(0), over the probability distribution for a muon and for a hydrogen atom. The stretch contribution dominates. The effect of muon motion on its methylene neighbor is small but significant. The effect of Mu motion on the more distant atoms is much smaller and similar to that due to H motion so the isotope effect for the *ortho*, *meta* and *para* H atoms is very small. 

The three experimental values given are for the lowest temperature reported for the gas phase muon values (313 K) and a solution value for 298 K. These would all be the same in the absence of zero point averaging and solvation effects. The difference Mu-H1' = 56.9 − 44.6 = 12.3 G is compared to the calculated value of 11.3 G given by the difference in the ZPC of 11.1 − (−0.2). No contribution from solvation is involved in this difference. The difference H1' – H1 for H1' in C_6_H_6_Mu and H1 (or H1') in C_6_H_7_ observed to be 44.57 − 48.03 = −3.46 G is computed to be −3.20 G. This experimental difference includes the solvent effect. This can be estimated by using the solvated value for C_6_H_6_Mu which is [5] 44.78 G to give 44.78 − 48.03 = −3.25 G. This value is given in Table 1. 

**Table 1:** Summary of calculations and comparison with experiment (all values in Gauss).

	**moved:**	**muon**		**hydrogen**
	**responding:**	**Mu**	**H1'**	**H1**
	Stretch	10.15	−0.23	2.88
	In Plane	0.65	−0.05	−0.01
	Out of Plane	0.33	0.08	0.13
	**Total ZPC:**	**11.12**	**−0.20**	**3.00**
EXPERIMENT:*	Gauss	**56.89**	**44.57**	**48.03**
EXP DIFFERENCE:	Gauss	12.32		−3.25 #
CALC DIFFERENCE:	Gauss	11.32		−3.20
OPTIMIZED a(R_e_):	***45.19***	**56.31**	**44.99**	**48.19**
RMSD & EXP-CALC :	**0.42**	0.58	−0.42	−0.16

* Taken from [6]. The C_6_H_7_ data in [6] is from [11]. # See text above.

Another way of making comparison of computed and experimental results is to determine what single value of a(0) for the H1 hydrogen atoms, the same for all three cases, will result in best agreement with experiment. The value found is 45.19 Gauss. With this value and the computed ZPCs the absolute observations are computed within 0.5 G on the average. The differences of this zero point correction calculation and experiment shown above are roughly equal to the reported experimental uncertainty of ca. 0.25 G. This good agreement is probably somewhat coincidental since solvent and temperature effects, although small, are measurable. 

**Figure 4:**
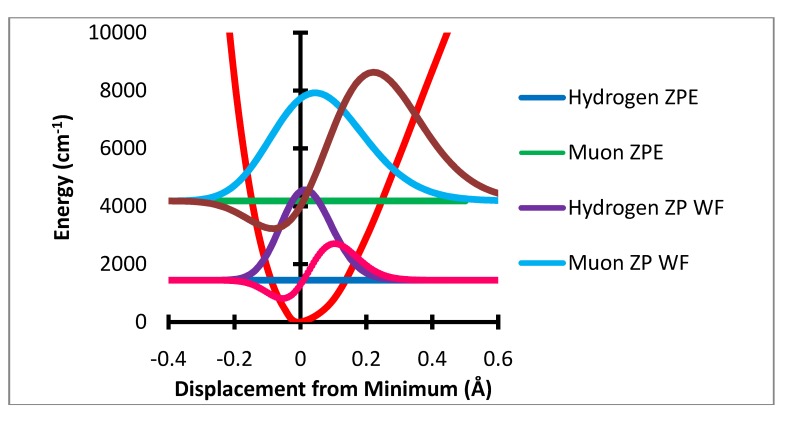
Stretching displacement potential (red curve) and probability distributions for H and Mu showing (dashed or dotted lines) the product of the probability times the variation of a(R-R_e_)-a(R_e_).

The ZPC correction is dominated by the stretching degree of freedom. The mean extension of the H atom from the minimum is 0.0217 Å while that for muonium is 0.0687 Å. The HFCC rises rapidly with increase in R-R_e_ In the present treatment the effect of the stretching motion is computed by multiplication of the variation of the HFCs, a(R-R_e_) – a(R_e_) by the probability that the bond length R is sampled by the normalized wavefunction. It should be noted that the effect of the change in bond length is only a small part of this change, ca. 3.5 G out of the 10 G total effect. The major net effect is due to the rapid rise of a(R-R_e_) –a(R_e_) with large positive values of R-R_e_ and the much greater sampling of this region by the muon radial motion. This is shown in Figure 4 where the product of the variation of a(R-R_e_)-a(R_e_) times the probability distribution is shown as the dashed lines. The integral of this function is the radial part of the ZPC for each case. The difference of the integrals is the isotope effect. It is clear that the largest effect comes from the region of R-R_e_ near 0.3 Å. 

The method used in previous treatments appears to be suspect. The procedure followed was: “A possible way of simulating this isotopic perturbation is by increasing the C-H bond length of H species, analogous to the muonic one, by 4.9% and keeping it fixed while the remaining geometry parameters are relaxed in a geometry optimization calculation using the MNDO (RHF version) semi-empirical program [14].” This would seem to be treating the muonium as if it was a very *heavy* atom.

Calculations of the ZPC for the HFCCs for the other H atoms of C_6_H_7_ have been made in this local mode treatment and were found to be much smaller than for the methylene H atoms. It was found that the bend contributions to this more distant effect were on the same order as the stretch contribution. This is also observed for the ^13^C-NMR H/D effect. By hypothesis of this local mode approach these will cancel in the difference between C_6_H_7_ and C_6_H_6_Mu, *i.e.*, the calculation of the isotope effect. It is reassuring in this respect that these effects are small. However the zero point correction may be a significant fraction of the HFCC since these are also small.

Despite the good agreement shown above, there are reasons to suspect the local mode treatment presented here. One of these is the fundamental issue that we have not checked the assumption that the effects of stretch and bend contributions are in fact additive. This was found to be the case for the ^13^C-NMR H/D effect. It has been argued that the isotope effect of replacement of H by Mu in C_6_H_7_ is larger for C_6_D_6_H *vs.* C_6_D_6_Mu [6]. This cannot be accounted for in our approach but seems to be based on only one data point.

A more obvious issue is that the shape of the potential energy in the region of large extension is important in obtaining the correct result. Our stretch potential is based on a computed B3LYP/6-311G(2d,2p) potential that is fit in its lower region to a Morse potential. The larger displacement part of the potential energy is not taken directly from the calculation due to incorrect dissociation behavior of DFT methods. Instead the asymptotic value is taken from a calculation on the deformed benzene plus the exact result for an H atom. This involves the energy difference between the optimized C_6_H_7_ open shell structure and a bent C_6_H_6_ structure. This dissociation energy and the shape of the potential as this limit is approached should be obtained from more advanced methods. This may be the origin of the 10% deficit of the computed value for the ZPC correction difference. Despite these reservations, the agreement observed between computed and observed effects supports several conclusions. 

## 3. Methods

The structure of C_6_H_7_ in C_2v_ symmetry is optimized using Gaussian 03 [17] B3LYP/6-311G(2d,2p). This is the starting point for the bond length and angle deformation calculations. The stretch, in-plane and out of plane deformations, in both positive and negative displacements away from the equilibrium position are made in small intervals with at least 5 points in each direction.

In performing the fit for the Morse potential representing the variation of energy with R, the large R part of the computation is discounted in favor of the known dissociation limit which is the energy obtained by the same computation for the structure made by removing one of the methylene hydrogen atoms from the optimized C_2v_ C_6_H_7_ structure to make a C_6_H_6_ with an out-of-plane CH bond. The hydrogen energy of minus 0.5 atomic units is added to this. The resulting Morse parameters R_e_ = 1.09Å, β = 2.617/Å and D_e_ = 18630 cm^−1^ in the designation and units as used for input for the program FGH [18,19]. The reduced mass used in an FGH calculation is 1.008 amu for H or 0.11345 amu for Mu [1]. The final results were computed with 300 points over a range adjusted to be sure that the integrand product (Figure 4) had dropped to zero.

The series of calculations at displaced geometry produces values for each of the hyperfine coupling constants. The values for each displacement minus the value at the equilibrium geometry, a(0), are plotted as a function of the displacement and the variation of each hyperfine constant with displacement is fit to a sixth order polynomial. This polynomial is then evaluated at the values of the displacement at which the corresponding numerical wavefunction was evaluated. These interpolated values of a(ξ) (ξ = δR, δθ or δφ) are then multiplied by the value of the normalized square of the wavefunction for H or Mu at the same value of the displacement (Figure 4) and the result summed.

## 4. Conclusions

It should first be noted that the difference in the experimental values in Table 1 of 56.89 (Mu), 44.57 (H1') of C_6_H_6_Mu and 48.03 for H1 of C_6_H_7_ can only be explained by inclusion of at least the zero point correction to the HFCC values as done here. No electronic structure calculation at any single geometry can account for the variation in these numbers. This is seen by comparison of results for the two isotopic species but a zero point correction is also potentially important in a quantitative treatment for the *ortho*, *meta* and *para* hydrogen atoms. 

One of the conclusions of this work is that, although it is the stretching degree of freedom dominates the zero point effect, it is not the change in bond length that is most important but rather the change in range of the stretching motion. This differs from the ^13^C-NMR H/D effect and is due to much larger mass ratio for this case. Another conclusion is that the value for the HFCCat the minimum energy position, a(0), that should be compared with a computation for an optimized geometry is 45.2 ± 0.6 G. The error limit range given is obtained by “correcting” each of the three experimental values by their respective zero point correction and taking the average and standard deviation. The resulting range encompasses both the experimental variation and the apparent 8% error in the zero point level shift difference Mu * vs.* H. 

**Figure 5:**
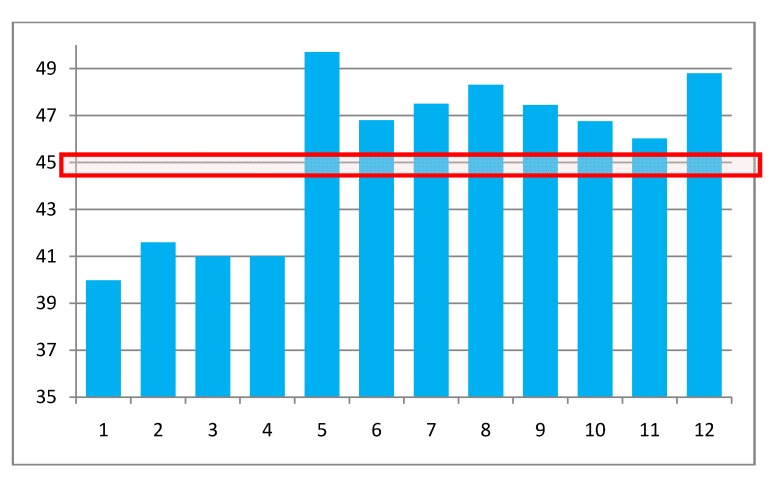
Graphical comparison of a(0) = a(R_e_,0,0) computed as indicated in Table 2. The numbers on the entries are in the right hand column of Table 2. The red box shows the “target” range of values.

This consensus value of 45.2 ± 0.6 G is compared to the a(R_e_) values computed by several methods and geometries in Table 2 and Figure 5. Entries 1 and 2 are from [20] and use a geometry from [21] based on an AM1 calculation; the other ten entries are from this work. Each entry is based on a geometry and a HFCC method at that geometry. For entries 3–5 the geometry is optimized with the same method used for the HFCC calculation. The other calculations use the indicated MP2/basis method indicated for optimization and the DFT/basis indicated for HFCC. 

**Table 2:** Comparison of vibrationally corrected HFCC for H(1) with computed a(0) values.

**Method**	**Basis set**	**a(0)**	
target value based on vibrationally corrected experiment:		**45.2 ± 0.6**	#
Geometry from AM1			
CCSD(T)	[9s,5p,1d]/(4s,2p,1d) C [4s,1p]/(2s,1p) H	39.98 *	1
CCSD	[9s,5p,1d]/(4s,2p,1d) C [4s,1p]/(2s,1p) H	41.60 *	2
optimized with method used for HFCC calculation			
CCSD (opt)	6-31G(2d,2p)	41.00	3
CCSD(opt)	6-311^++^G(2d,2p)	41.00	4
DFT/B3LYP(opt)	various up to 6-311^++^G(3d,3p)	49.2–50.2	5
optimized with MP2/6-311^++^G(2d,2p)			
DFT/B3LYP	6-31G(2d,2p)	46.80	6
DFT/B3LYP	6-311 G(2d,2p)	47.50	7
optimized with MP2/6-311++G(3d,3p)			
DFT/B3LYP	cc-pVQZ	48.31	8
DFT/B3LYP	aug-cc-pVQZ	47.45	9
DFT/B3LYP	6-311^++^G(3d,3p)	46.76	10
DFT/B3LYP	6-311^++^G(3df, 3pd)	46.02	11
DFT/PBE0	EPR basis set III (ref 21)	48.80	12

These same results are compared in a graphical format in Figure 5 to each other and to the “target” value that would result in agreement with experiment when the zero point corrections of Table 1 are added. It is seen that all of the correlated CCSD values, entries 1–4, are too low by a large margin. This is also the case for MP2 HFCC values. The DFT/B3LYP values comprising entry 5 used this method for both geometry and HFCC calculations. The range of a(0) values, 49.2 to 50.2, representing various basis sets, are all considerably too high. Entries 6–12 are all DFT results based on MP2 geometries. The large conventional basis set, entry 11, differs from the ZPL corrected estimate by 1.25 times the estimated standard error of this extracted value, *i.e.*, essentially in agreement. Use of the PBE functional and the EPR-III basis set [22] results in a value considerably too large. The fact that these considerations discriminate so clearly among computed results shows their utility. 

The *ortho*, *meta* and *para* hydrogen atoms of C_6_H_7_ have HFCC values very similar to those for C_6_H_6_Mu. This absence of an isotope effect does not necessarily mean that the observed values are unaffected by zero point averaging but only that the large effect at the methylene position are not transmitted to the other centers. The computed zero point corrections using the same local approximation are somewhat too small to result in agreement with experiment but they are in the correct direction. 

The HFCC values obtained using the method and basis set “11” that best fits the target methylene value results in static a(0) values that are strongly correlated with the experimental values but are about 20% too small in an absolute sense (viz, *ortho:* −9.13 exp.; −7.30 calc.; *meta:* 2.65 exp.; 1.66 calc.; *para:* −13.56 exp.; −11.19 calc.). The RMS deviation is 1.1 Gauss. The PBE0 EPR III results are, on average 30% too large. Method “10” which is the second closet match to the target a(0) value gives an RMS deviation of only 0.22 Gauss which is probably within experimental error. The diagonal (*i.e*., only the H in question moves) zero point corrections for the *ortho*, *meta* and *para* hydrogen atoms are −0.265 G, +0.154 G and −0.300 G, respectively. Addition of these corrections results in an RMS deviation of 0.91 G for set 10 and of 0.14 G for set 10. The fact that the a(0) values are slightly too small in absolute value is thus in the right direction.

The method introduced here for the computation of HFCCs of muon containing radicals is an outgrowth of our work on ^13^C-NMR isotopic shift computations. It is a specific case of a more general treatment applied to EPR HFC’s of the parent radicals [22]. This more general normal-mode based method may result in small individual contributions giving a cumulative effect that is in agreement with experiment. Further analysis will include automation of this zero point correction procedure and further exploration of the electronic structure methodology that is needed to provide agreement with experiment. The local mode approach permits treatment of large systems.
